# Fabrication of Flexible pH-Responsive Agarose/Succinoglycan Hydrogels for Controlled Drug Release

**DOI:** 10.3390/polym13132049

**Published:** 2021-06-22

**Authors:** Yiluo Hu, Yohan Kim, Inki Hong, Moosung Kim, Seunho Jung

**Affiliations:** 1 Center for Biotechnology Research in UBITA (CBRU), Department of Bioscience and Biotechnology, Konkuk University, Seoul 05029, Korea; lannyhu0806@hotmail.com (Y.H.); shsks1@hanmail.net (Y.K.); 2 Covergence Technology Laboratory, Kolmar Korea, 61, Heolleung-ro-8-gil, Seocho-gu, Seoul 05029, Korea; inkiaaa@kolmar.co.kr; 3 Macrocare, 32 Gangni 1-gil, Cheongju 28126, Korea; rnd@macrocare.net; 4 Center for Biotechnology Research in UBITA (CBRU), Department of Systems Biotechnology, Institute for Ubiquitous Information Technology and Applications (UBITA), Konkuk University, Seoul 05029, Korea

**Keywords:** hydrogels, agarose, succinoglycan, drug delivery, pH-responsive

## Abstract

Agarose/succinoglycan hydrogels were prepared as pH-responsive drug delivery systems with significantly improved flexibility, thermostability, and porosity compared to agarose gels alone. Agarose/succinoglycan hydrogels were made using agarose and succinoglycan, a polysaccharide directly isolated from *Sinorhizobium meliloti*. Mechanical and physical properties of agarose/succinoglycan hydrogels were investigated using various instrumental methods such as rheological measurements, attenuated total reflection–Fourier transform infrared (ATR-FTIR) spectroscopic analysis, X-ray diffraction (XRD), and field-emission scanning electron microscopy (FE-SEM). The results showed that the agarose/succinoglycan hydrogels became flexible and stable network gels with an improved swelling pattern in basic solution compared to the hard and brittle agarose gel alone. In addition, these hydrogels showed a pH-responsive delivery of ciprofloxacin (CPFX), with a cumulative release of ~41% within 35 h at pH 1.2 and complete release at pH 7.4. Agarose/succinoglycan hydrogels also proved to be non-toxic as a result of the cell cytotoxicity test, suggesting that these hydrogels would be a potential natural biomaterial for biomedical applications such as various drug delivery system and cell culture scaffolds.

## 1. Introduction

Many studies have been developed for drug delivery systems using liposomes, microspheres, and nanoparticles [1]. Among them, hydrogels are attracting much attention as one of the promising drug delivery systems because of their hydrophilicity, softness, biocompatibility and high absorption rate. Additionally, natural polysaccharides such as cellulose, alginate, starch, and gellan have been explored as biomaterials for drug delivery hydrogels due to their non-toxicity, availability, stability, and biodegradability [2]. Especially, such polysaccharides contain different functional groups such as hydroxyl, amino, carboxylic acid, and aldehydes, making them ideal for stimuli-responsiveness and diverse chemical conjugations [3]. Accordingly, polysaccharide-based hydrogels have been actively used in various fields of cosmetics, pharmaceuticals, and regenerative medicine [4]. 

**Figure 1:**
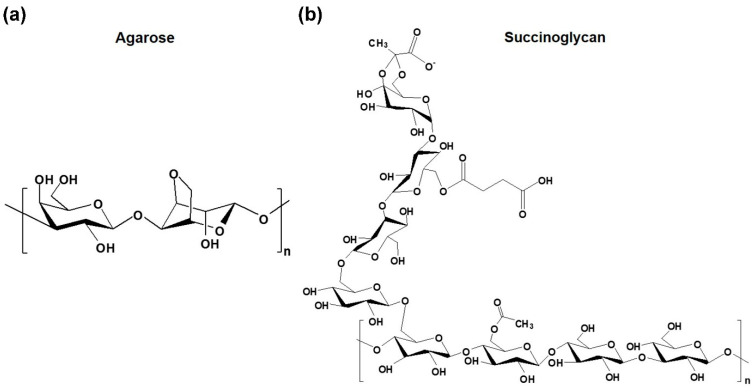
Chemical structures of (**a**) agarose and (**b**) succinoglycan.

Agarose is a type of natural polysaccharide produced by red algae, which is composed of 1‚4-linked 3‚6-anhydro-alpha-l-galactose and 1‚3-linked β-d-galactose derivatives, as shown in Figure 1a [5]. Pure agarose has the constant thermo-responsive behavior of undercooling and its gel–sol transition occurs depending on the temperature [6]. This water-soluble polysaccharide generally forms a network structure at low temperatures, creating a water-insoluble special gel-type structure [5,6]. Agarose hydrogels have attracted some attention for tissue engineering [7], drug delivery [8], and rapid wound healing [9]. This is due to their good biocompatibility, thermo-reversibility, stability in vivo systems, and high mechanical properties. However, natural agarose hydrogels have some disadvantages such as hardness, brittleness, and slow decomposition rates. To overcome these shortcomings, various chemical functional groups such as amine, carboxyl, and even β-cyclodextrin were introduced into agarose [5,9,10]. In other studies, agarose was sometimes used for the preparation of interpenetrating hydrogels with other biopolymers such as konjac glucomannan, salecan, and chitosan to improve the physical properties of hydrogels without cross-linking agents [11,12,13]. However, in agarose/chitosan hydrogels, organic solvent was used for the preparation process of hydrogels due to the low aqueous solubility of chitosan [14]. Most other natural interpenetrating biopolymers are neutral, so they did not show clear pH-responsive drug delivery effects caused by pH-dependent differential swelling behavior.

Succinoglycan is an acidic natural polysaccharide isolated from microorganisms such as *Agrobacterium tumefaciens* and *Sinorhizobium meliloti*, playing an important role in bacterial infection of legumes [15,16]. As shown in Figure 1b, succinoglycan consists of seven glucose residues and one galactose residue with β-1‚3, β-1‚4, and β-1‚6-linked subunits, including pyruvate, succinate, and acetate groups, and is known to have a double helix structure at 25 °C [17,18]. It possesses excellent water solubility and good biodegradability and biocompatibility, facilitating its commercial application in food, cosmetics, pharmaceuticals, emulsifiers, etc. [19,20,21]. In particular, succinoglycan has a high viscosity and thermal stability due to its unique structural properties [22,23]. Recently, a study using a gel–sol transition induced by treating a hydrogel made of ferric ion (Fe^3+^) and succinoglycan with a reducing agent for drug delivery was published [24]. However, the influence of succinoglycan on the properties of agarose hydrogels has not been reported so far.

The purpose of this study is to prepare functional agarose/succinoglycan composite hydrogels, allowing both pH-dependent drug delivery and scaffolds for cell culture. We used microbial succinoglycan, isolated directly from *Sinorhizobium meliloti*, adding succinoglycan to the agarose to change its intrinsic rigid and brittle physical properties. To this end, microbial succinoglycan that has high intrinsic viscosity and thermal stability was used to improve the rheological properties of agarose [23,25]. Various mechanical, rheological, and physical properties of those agarose/succinoglycan hydrogels were investigated using a rheometer, attenuated total reflection–Fourier transform infrared spectroscopy (ATR-FTIR), X-ray diffraction (XRD), and field emission scanning electron microscopy (FE-SEM). We also investigated the pH-dependent drug release property as well as cell cytotoxicity for these composite hydrogels.

## 2. Materials and Methods

### 2.1. Materials

Purified succinoglycan was prepared and its average molecular weight was 35 KDa [24]. Agarose (EEO value, 0.05–0.13; sulfate, <0.15%) was purchased from BioPure (Seoul, South Korea). Ciprofloxacin (CPFX) was purchased from Sigma-Aldrich (St. Louis, MO, USA). Swelling tests and drug release tests were studied both in phosphate-buffered saline (PBS, pH 7.4) and 0.1 N hydrochloric acid solution (HCl, pH 1.2). Sodium phosphate dibasic (Na_2_HPO_4_) and HCl were purchased from Daejung (Siheung, Korea). Potassium dihydrogen phosphate (KH_2_PO_4_), potassium chloride (KCl), and sodium chloride (NaCl) were purchased from Samchun Pure chemicals (Anyang, Korea). All other chemicals were of analytical grade and used without further purification.

### 2.2. Preparation of Agarose/Succinoglycan Hydrogels

To fabricate agarose/succinoglycan hydrogels, a 2% (*w*/*v*) stock solution of agarose and succinoglycan was prepared in advance. Briefly, 200 mg of agarose powder was suspended in distilled water (10 mL) and then autoclaved at 100 °C for 10 min to obtain a completely dissolved solution. In addition, 200 mg of succinoglycan was dissolved in distilled water (10 mL) and stirred to obtain a solution. The agarose solution (2–10 mL, 2%, *w*/*v*) and the succinoglycan solution (2–8 mL, 2%, *w*/*v*) were mixed together and stirred on a 70 °C hot plate for 15 min under a stirring condition of 400 rpm. The mixture was then maintained at 4 °C overnight to induce gelation. A detailed description of agarose and succinoglycan proportions is listed in Table 1. The prepared hydrogels were labeled AG10, AG8/SG2, AG6/SG4, AG4/SG6, and AG2/SG8, respectively.

### 2.3. Rheological Measurements

The rheological experiments were performed with a DHR-2 rheometer (TA Instruments, Waltham, MA, USA) to measure dynamic strain sweeps, oscillatory angular frequency sweeps, and dynamic temperature sweeps. To determine the limitation of the linear viscoelastic region, a strain sweep experiment was performed from 0.1% to 100% at a frequency of 1 Hz. The angular frequency sweep was tested by applying 1.0% constant strain from 0.1 to 100 rad/s at 25 °C. Temperature ramp tests were performed with a 5% strain at an angular frequency of 1 Hz with a cooling cycle of 80 to 20 °C and a heating cycle of 20 to 100 °C. The heating and cooling cycle temperature rates were set at 1 °C/min.

### 2.4. Attenuated Total Reflection–Fourier Transform Infrared (ATR-FTIR) Spectroscopy

ATR-FTIR spectroscopy was carried out on an ATR-FTIR spectrometer (Spectrum Two FT-IR, Perkin Elmer, Waltham, Massachusetts, USA) equipped with a PIKE MIRacle ATR accessory. Prior to measurement, all samples were prepared by lyophilization. The hydrogel samples were recorded by ATR-FTIR spectrometer in the range from 500 to 4000 cm^−1^ at a resolution of 1 cm^−1^ using 8 scans. After that, data analysis was performed using Spectrum software.

### 2.5. X-ray Diffraction (XRD) Measurements

The crystal structure of the agarose, succinoglycan and prepared agarose/succinoglycan hydrogels was investigated using an X-ray analytical instrument (Rigaku SmartLab, Japan) equipped with a HyPix-3000 detector. X-ray diffraction (XRD) patterns within the range 2θ = 10–60° were examined by using Cu Kα under the tube voltage of 30 kV and the tube current of 20 mA.

### 2.6. Field Emission Scanning Electron Microscopy (FE-SEM) Analysis

The morphology of agarose/succinoglycan hydrogels was analyzed by using FE-SEM (Hitachi S-4700, Tokyo, Japan) at an acceleration voltage of 5 kV. All hydrogels were lyophilized and coated with platinum layer at 30 w for 60 s in a vacuum. For energy-dispersive analysis (EDS), FE-SEM (AURIGA, Carl Zeiss, Germany) equipped with an Energy selective Backscattered (EsB) detector was used to measure the uniformity of the elements in the hydrogel. 

### 2.7. Swelling Tests

The swelling behavior of agarose/succinoglycan hydrogels was explored at pH 1.2 and pH 7.4 buffers at 37 °C and swelling phases were carried out for 6 h. Each of the lyophilized agarose/succinoglycan hydrogels was immersed in pH 1.2 and pH 7.4 buffer, respectively. Then, the swollen hydrogel was taken out of the buffer and weighed after removing excess surface solution by using filter paper. The swelling ratio was obtained based on the following equation [26]:

$$\mathrm{Swelling}\;\mathrm{ratio}\;\left(\%\right)=\frac{w_{s}-w_{d}}{w_{s}}\times 100$$

where W_S_ and W_d_ represent the weight of the swollen and dried hydrogels, respectively. Each experiment was conducted three times.

### 2.8. In Vitro Drug Loading and Release Studies

To estimate the effect of succinoglycan on drug release of agarose hydrogels, the cumulative release of CPFX was studied using AG10 and AG4/SG6 hydrogels, which were selected according to swelling tests and morphology studies. Firstly, CPFX (1 mg) was added to 600 µL of succinoglycan solution (2%, *w*/*v*) and stirred to prepare a homogeneous solution. Subsequently, the succinoglycan mixed with CPFX solution was stirred with 400 µL of agarose solution (2%, *w*/*v*) at 70 °C. CPFX-loaded AG10 and AG4/SG6 hydrogels were dried in vacuum oven at 50 °C overnight [27]. The dried CPFX-loaded hydrogel was dissolved in release buffer and stirred for 6 h. Then, its suspension was passed through a filter and analyzed with UV spectrophotometer. The CPFX loading amount and encapsulation efficiencies were evaluated with the following equations, respectively [24]:

$$\mathrm{Loading}\;\mathrm{amount}\;\mathrm{of}\;\mathrm{the}\;\mathrm{CPFX}=\frac{\mathrm{Extract}\;\mathrm{of}\;\mathrm{CPFX}}{\mathrm{Weight}\;\mathrm{of}\;\mathrm{hydrogels}}$$

$$\mathrm{Encapsulation}\;\mathrm{efficiency}\;\left(\%\right)=\frac{\mathrm{Loading}\;\mathrm{amount}\;\mathrm{of}\;\mathrm{CPFX}}{\mathrm{Weight}\;\mathrm{of}\;\mathrm{the}\;\mathrm{theoretical}\;\mathrm{CPFX}}$$

Based on this equation, each of the loading amounts of CPFX in AG10 and AG4/SG6 hydrogels was 12 mg/g and 14 mg/g, respectively. Then, the CPFX-loaded AG10 and AG4/SG6 hydrogels were immersed in 20 mL buffer (pH = 1.2 and 7.4) at 37 °C with 100 rpm of constant stirring. At specific interval points, 500 µL of CPFX released medium was determined at 276 nm. The cumulative amount of CPFX was calculated with the following equation:

$$\mathrm{Cumulative}\;\mathrm{amount}\;\mathrm{of}\;\mathrm{the}\;\mathrm{CPFX}=C_{n}V+\overset{i=n-1}{\underset{i-1}{\sum }}C_{i}V_{i}$$

*V* is the release of the medium volume, *V_i_* is the sampling volume, and *C_n_* and *C_i_* are the CPFX concentrations in the release medium and the aliquots. All measurements were repeated three times.

### 2.9. In Vitro Cytotoxicity

Cytotoxicity of the agarose/succinoglycan hydrogels was evaluated by WST-1 assay tests. The human embryonic kidney 239 (HEK-293) cells were obtained from the Korean Cell Line Bank (South Korea) and they were cultured in minimum essential medium (MEM, WELGENE, Gyeongsan-si, South Korea) supplemented with 10% fetal bovine serum (FBS) and 1% antibiotics (100 U/mL penicillin and 100 g/mL streptomycin). The cultured cells were maintained in a humidified incubator with 95% air and 5% CO_2_ atmosphere at 37 °C. Thereafter, 10 mg of lyophilized hydrogel sample stored in a 24-well plate containing 1 × 10^5^ cells/well was added to the wells and then the plate was incubated for 48 h. Subsequently, WST-1 assay reagent was added to each well and reacted for 4 h in the incubator, and then the absorbance was measured using microplate reader at 450 nm. Cell viability was calculated using the following formula:

$$\mathrm{Cell}\;\mathrm{viability}\;\left(\%\right)=\frac{\mathrm{Absorbance}\;\mathrm{of}\;\mathrm{cells}\;\mathrm{with}\;\mathrm{hydrogel}}{\mathrm{Absorbance}\;\mathrm{of}\;\mathrm{negative}\;\mathrm{control}\;\mathrm{cells}}$$

All assays were repeated three times for each sample.

## 3. Result and Discussion

### 3.1. Rheological Analysis

**Figure 2:**
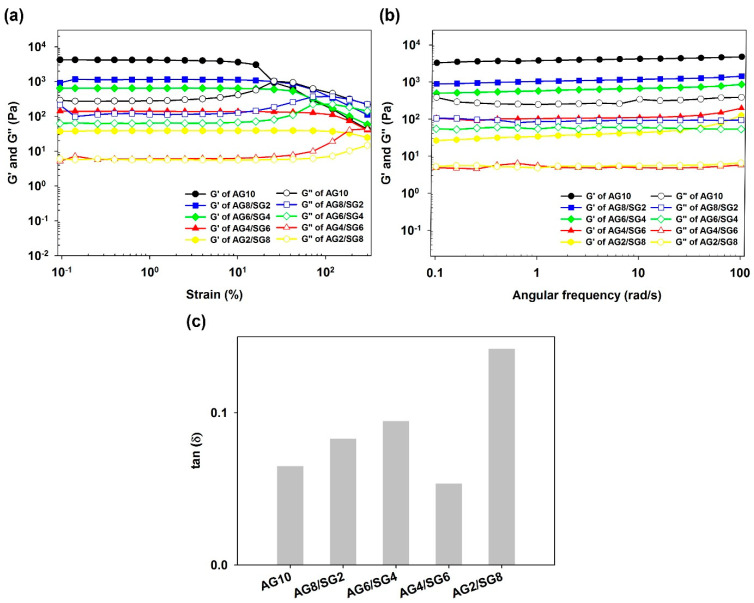
Storage modulus (G’) and loss modulus (G”) of (**a**) strain sweep for agarose and agarose/succinoglycan hydrogels at frequency of 1 Hz at 25 °C. (**b**) Angular frequency dependencies of G’ and G” for hydrogels with different agarose/su- ccinoglycan ratios. (**c**) The loss tangent (tan δ = G”/G’) of agarose/succinoglycan hydrogels when the angular frequency is 1 rad/s.

The strain sweep analysis was performed to evaluate the viscoelastic properties of hydrogels at different mass ratios [11]. The storage modulus (G’) and loss modulus (G”) characterize the elastic and viscosity properties, respectively. The values of G’ and G” were constant within 10% strain, indicating the elastic structure of hydrogel remained stable over the 0.1–10% range of strain. The cross-over point of the AG10 hydrogel was observed at the 26% oscillatory strain, but it was observed that increasing the concentration of succinoglycan in the composite hydrogels also increased the strain value at the cross-over point of the hydrogels (Figure 2a). These results probably indicated that hydrogen bonding interactions between the agarose and succinoglycan would reduce the aggregation of the helical structures of the agarose, thereby forming more flexible hydrogels [11].

Angular frequency sweep tests are normally used to determine the structural integrity and mechanical strength of a material. The G’ and G” values for the hydrogels as a function of angular frequency are shown in Figure 2b while keeping the 1% strain constant without the effect of strain on the measured dynamic modulus. Within each frequency range tested (0.1–100 rad/s), G’ was always greater than G″ and both values did not change significantly with increasing frequency. It was confirmed that the elastic property dominated the viscous property. Increasing the succinoglycan concentration in the agarose/succinoglycan hydrogels decreased the dynamic modulus, making it more evident that a more flexible network hydrogel was produced. Among these flexible gel conditions, the loss tangent was calculated to select a hydrogel with proper physical properties to be used for swelling and drug delivery. The loss tangent (G”/G’, tan δ) is a measure of the relative contribution of the viscous component to the mechanical properties of a hydrogel when the frequency is a function of 1 rad/s. For weak gels, tan δ values range from 0.1 to 1.0, and for conventional gels tan δ values are less than 0.1 [28]. As shown in Figure 2c, the tan δ values of AG10 and AG8/SG2, AG6/SG4, AG4/SG6, and AG2/SG8 hydrogels were 0.065, 0.083, 0.095, 0.054, and 0.142, respectively. As the ratio of succinoglycan increased, tan δ also increased, and in the case of AG4/SG6, the tan δ value was observed to be smaller than that of agarose, suggesting that the gel properties of AG4/SG6 may be better than those of agarose gel. According to the results of this experiment, cases with tan δ values greater than 0.1 resulting in weak gels were excluded from subsequent studies. 

### 3.2. Sol–Gel Transition Studies

**Figure 3:**
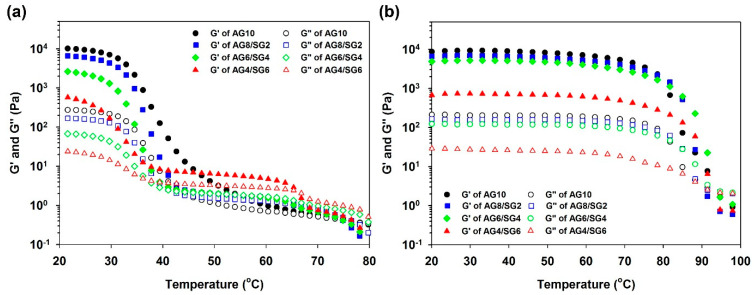
Temperature dependence of G’ and G” during (**a**) cooling and (**b**) heating process for agarose/succinoglycan hydrogels.

The temperature dependence of the G’ and G” while cooling agarose and agarose/succinoglycan hydrogels from 80 to 20 °C or heating from 20 to 100 °C is shown in Figure 3a,b, respectively. The cross-over point of each modulus in the sol–gel and gel–sol curves was determined as the gelling and melting point. The viscoelastic measurements for AG10 hydrogel showed the maximum storage modulus at the lowest temperature, but the storage modulus (G’) gradually decreased as the proportion of succinoglycan increased. In Figure 3a, the cooling curve of AG10 initially predominated in its viscous properties and then showed a rapid increase in G’ modulus at a cross-over point of 65 °C. This is probably due to the fact that the agarose molecule exists in a random coil state at a high temperature, and a double helix is formed after the gel point is formed [29,30]. In AG8/SG2 and AG6/SG4 hydrogels, it was lowered to 43 and 40 °C, respectively. The reason for this is probably that in the case of AG8/SG2 and AG6/SG4, the formation of the gel network was limited because the concentration of succinoglycan was not sufficient to provide a continuous network with agarose [28].

In the case of the AG4/SG6 hydrogel, the gelation temperature was observed at 66 °C, and the viscoelastic modulus value was observed to increase rapidly at about 40 °C. Compared with the cases of AG8/SG2 and AG6/SG4, AG4/SG6 exhibited elasticity during a period in which G’ rapidly increased at a gel point similar to agarose. This is probably because at this ratio succinoglycan and agarose are effectively converted into helices from random coils through optimal van der Waals and/or hydrogen bondings to form a hydrogel network [30]. In addition, this result suggests that AG4/SG6 is a hydrogel with lower mechanical strength but higher flexibility than AG8/SG2 and AG6/SG4 (Figure 2). On the other hand, the melting temperature (T_m_) was similar for all hydrogels, as shown in Figure 3b, showing that the succinoglycan contained in the hydrogel induced high temperature stability of the composite hydrogels. The AG10 hydrogel used as a control showed two steps in the heating process, but the agarose/succinoglycan hydrogels showed one step downward curve. This suggested that unlike xylan or carrageenan shown in other studies [31,32], succinoglycan formed a hydrogel through an agarose-like network. In addition, it was shown that AG4/SG6 had the highest melting point, which suggested that this hydrogel would be cross-linked with the highest binding energy.

### 3.3. Attenuated Total Reflection–Fourier Transform Infrared (ATR-FTIR) Spectra Analysis 

**Figure 4:**
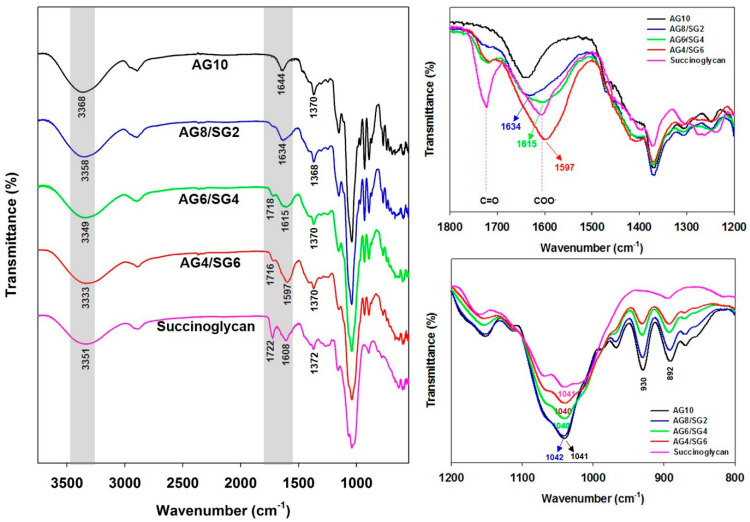
ATR-FTIR spectra of agarose/succinoglycan hydrogels and pure succinoglycan.

To understand the molecular interactions between agarose and succinoglycan in the composite hydrogels, ATR-FTIR spectroscopy was used to confirm the structure of agarose/succinoglycan hydrogels in Figure 4. The ATR-FTIR spectrum of pure AG10 hydrogel showed absorption peaks due to −OH stretching at 3368 cm^−1^, 2895 cm^−1^ for −CH_2_ stretching, 1370 cm^−1^ for C−C bending, and 1041 cm^−1^ for C−O stretching. The absorption bands at 929 cm^−1^ and 890 cm^−1^ were ascribed to 3,6-anhydrogalactose C−O−C vibration of the bridge and anomeric C−H of residual carbon, respectively [33,34]. The succinoglycan showed absorption peaks at 3351 cm^−1^, 2893 cm^−1^, and 1041 cm^−1^ corresponding to −OH, −CH_2_, and C−O stretching, respectively [24]. The absorption peak at 1722 cm^−1^ was associated with C=O stretching of the acetyl ester [35]. The absorption peaks observed at 1608 cm^−1^ and 1372 cm^−1^ are assigned to asymmetric and symmetric COO^−^ stretching vibration of the succinate and pyruvate acid groups [36]. However, in the case of agarose/succinoglycan hydrogels, as the ratio of succinoglycan increased, the −OH stretching vibration peak observed at 3351 cm^−1^ was shifted to 3358 cm^−1^ (AG8/SG2), 3349 cm^−1^ (AG6/SG4), and to 3333 cm^−1^ (AG4/SG6), respectively. This result was attributed to the formation of hydrogen bondings between the −OH groups of agarose and succinoglycan [13]. In addition, the COO^−^ peak of succinoglycan was shifted from 1608 to 1597 cm^−1^ in AG4/SG6 hydrogels, indicating the intermolecular hydrogen bondings between the carboxyl groups of succinoglycan and the hydroxyl groups of agarose. In addition, it was observed that the absorption peak of −C=O of succinoglycan, which did not appear in AG10, appeared and increased as the succinoglycan content of composite hydrogels increased. This confirmed the successful preparation of agarose/succinoglycan hydrogels. On the other hand, the decrease in peak intensities of 930 cm^−1^ and 892 cm^−1^ in the AG10 spectrum indicated that successful network formation was induced in agarose/succinoglycan hydrogels, probably due to the intermolecular interactions such as hydrogen bondings and van der Waals interactions [35,36]. 

### 3.4. X-ray Diffraction (XRD) Analysis

**Figure 5:**
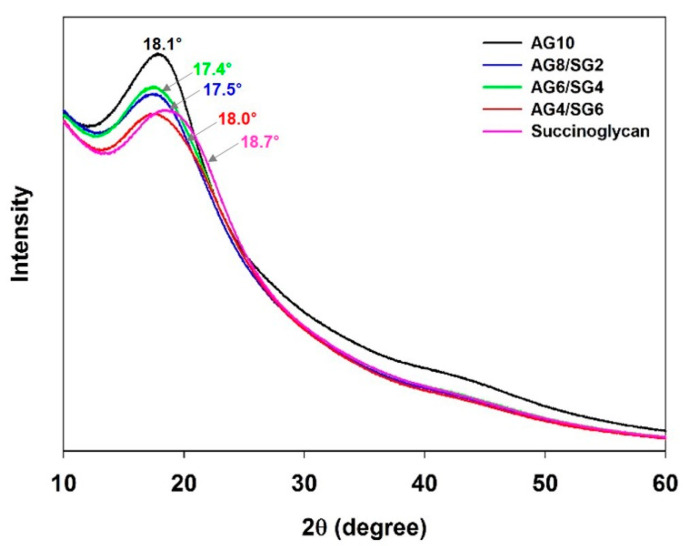
Changes in XRD patterns due to agarose/succinoglycan hydrogel formation by addition of succinoglycan.

The structure of prepared agarose/succinoglycan hydrogels was examined using the X-ray diffraction (XRD) technique. The results of XRD patterns of the AG10, succinoglycan, and agarose/succinoglycan hydrogels are shown in Figure 5. The diffraction pattern for AG10 showed a broad peak at 2θ = 18.1° because it did not crystallize during gelation [37]. The succinoglycan also displayed a broad peak at 2θ = 18.7°. However, AG8/SG2, AG6/SG4, and AG4/SG6 hydrogels showed lower and shifted diffraction peaks at angles of 17.5, 17.4, and 18.0 on the 2θ scale compared to agarose (AG10). The intensity reduction in agarose/succinoglycan hydrogels would indicate that succinoglycan could interfere with the ordering of the composite hydrogels. Similar changes of XRD patterns were reported for agarose-based composite hydrogel formation such as salecan, hyaluronic acid, and konjac glucomannan [11,12,37]. This phenomenon also indicated that the succinoglycan could be uniformly dispersed in the agarose/succinoglycan hydrogels to form a composite network.

### 3.5. Field Emission Scanning Electron (FE-SEM) Micrograph Analysis

**Figure 6:**
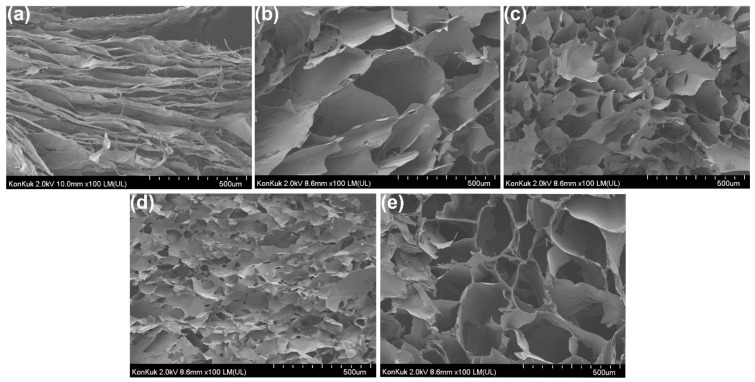
FE-SEM images illustrating the microstructures of cross-sectioned hydrogels: (**a**) Succinoglycan; (**b**) AG4/SG6; (**c**) AG6/SG4; (**d**) AG8/SG2; and (**e**) AG10.

To see the porosity and properties of the hydrogel structure, the morphological characteristics of the agarose/succinoglycan hydrogels were analyzed by scanning electron microscopy. The SEM image showed a cross-sectional micrograph of AG10 and agarose/succinoglycan hydrogels after introducing different concentrations of succinoglycan. As can be seen in Figure 6a, the hydrogels composed only of succinoglycans exhibited broad layers or sheets. Figure 6b (AG4/SG6) showed large pores probably due to network formation inside of the composite hydrogel. As the ratio of agarose increased to 60% (AG6/SG4, Figure 6c), and 80% (AG8/SG2, Figure 6d), the pore size decreased, respectively. These results would suggest that agarose chains could induce self-aggregation [13] and that those structures would be entrapped into succinoglycans of the composite hydrogels. On the other hand, the AG10 hydrogel composed only of agarose showed relatively large pores (Figure 6e). Since the pore size of a hydrogel generally determines the swelling rate and drug encapsulation potential, AG4/SG6 hydrogel would have enhanced the swelling rate and drug encapsulation potential compared with the others. This increased pore size of AG6/SG4 hydrogels also paralleled the observations in the lowest loss tangent (tan δ = G”/G’) of agarose/succinoglycan hydrogels in Figure 2. EDS mapping image analysis showed that the agarose/succinoglycan hydrogels had a uniform color distribution of carbon and oxygen elements. The EDS spectrum of each hydrogel showed only 70% carbon and 30% oxygen, and no other impurities were seen, suggesting the hydrogel homogeneity (Figures S1–S4). 

### 3.6. Swelling Behavior of Agarose/Succinoglycan Hydrogels

**Figure 7:**
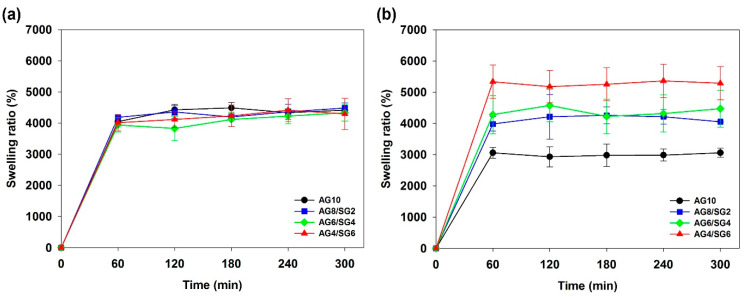
Swelling ratio curves for agarose/succinoglycan hydrogels in buffer (**a**) pH = 1.2 and (**b**) pH = 7.4 solution at 37 °C.

The degree of hydrogel swelling is the critical parameter that can control the release pattern of solvents and drugs in polymer networks [38]. In addition, many types of hydrogels have a variable swelling rate depending on the pH of the aqueous medium [3,39,40]. The cumulative release of CPFX was studied using AG10 (control) and AG4/SG6 hydrogels, where AG4/SG6 was selected due to its lowest loss tangent (tan δ = G”/G’) and highest swelling ratio. The swelling properties of agarose/succinoglycan hydrogels were investigated in different media of pH 1.2 and pH 7.4 at 37 °C. As shown in Figure 7, all hydrogel samples expanded rapidly at first and represented stable swelling equilibrium after 1 h. This is because water molecules are attracted to the OH groups of hydrophilic agarose and succinoglycan and diffuse into the hydrogel to reach final equilibrium [12]. At pH 1.2, there was no difference in the swelling percentage of agarose/succinoglycan hydrogels (Figure 7a), but it was observed that the swelling rate gradually increased at pH 7.4 as the succinoglycan proportion in the agarose/succinoglycan hydrogels increased (Figure 7b). Since succinoglycan has a pK_a_ of 3.8, the carboxyl group exists in a protonated form (−COOH) at pH 1.2 and a deprotonated form (−COO^−^) at pH 7.4 [41]. Therefore, in acidic conditions (pH 1.2), the hydrophilic groups of the agarose/succinoglycan hydrogel network became protonated to form intermolecular hydrogen bondings between the agarose and succinoglycan, thereby interfering with molecular interactions with water. On the contrary, in alkaline conditions (pH 7.4), carboxyl groups become deprotonated as carboxylate anions, so electrostatic and hydrogen bondings with water molecules are actively formed, resulting in the expansion of the gel network and surface area as a swelling effect. Therefore, it was shown that agarose/succinoglycan (AG4/SG6) hydrogels could be successfully used as a drug delivery carrier that exhibits pH-dependent drug release behavior as a composite hydrogel.

### 3.7. In Vitro Ciprofloxacin Release Study

**Figure 8:**
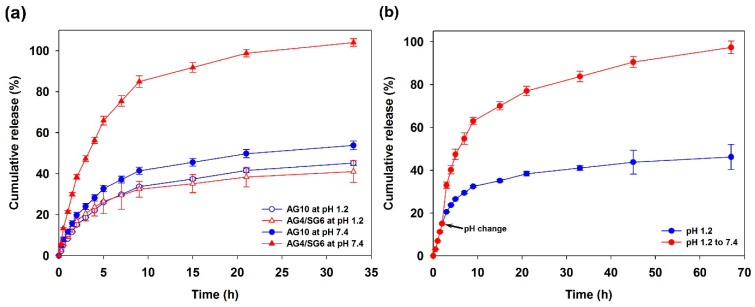
CPFX cumulative release graphs of AG10 and AG4/SG6 hydrogels loaded with CPFX in (**a**) pH media (pH 1.2 and 7.4) at 37 °C and (**b**) under pH change from 1.2 to 7.4 at 2 h.

Ciprofloxacin (CPFX) with high thermal stability was selected as a model for drug release studies using agarose/succinoglycan hydrogels [42]. CPFX is an antibacterial fluoroquinolone used in eye drops and to treat bacterial infections or ulcers in the eye [39]. As shown in Figure 8a, the CPFX-loaded agarose hydrogel (AG10) released the drug after 9 h at a similar rate, 45% and 53%, at pH 1.2 and pH 7.4, respectively. This is because the natural agarose hydrogel would control the diffusion phenomena that occurred after the initial burst release [8]. However, the agarose/succinoglycan (AG4/SG6) hydrogels with CPFX showed a significant difference between pH 1.2 and pH 7.4, where all of the CPFX was released within 33 h at pH 7.4. The low swelling rate of the acidic medium could limit drug release from the hydrogels; however, at alkaline pH, the composite hydrogels could expand rapidly, significantly increasing drug diffusion from the matrix. In addition, as a result of investigating the release rate when the pH changes instantaneously to 7.4 after 2 h starting at pH 1.2, it was observed that only about 45% was released in the case of pH 1.2, but all drugs were released after 65 h in the case of pH 7.4 (Figure 8b). This experiment was able to clearly demonstrate the characteristic pH-responsive drug release behaviors of agarose/succinoglycan hydrogels.

### 3.8. Cytotoxicity of Agarose/Succinoglycan Hydrogels

**Figure 9:**
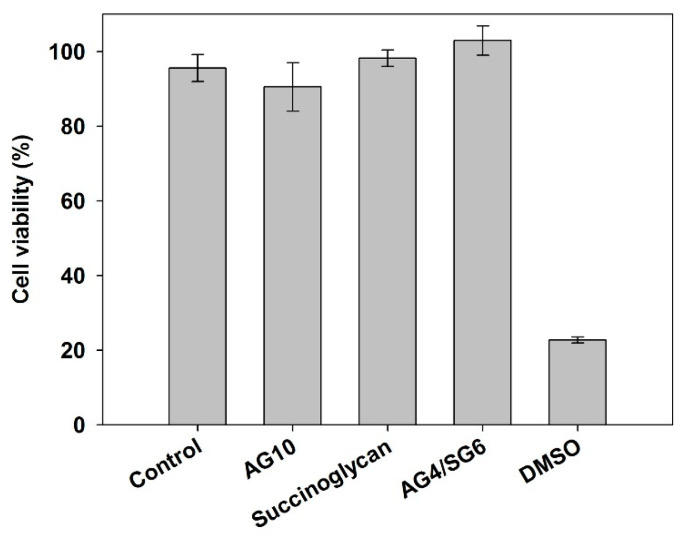
HEK-293 cell viability tests of control, succinoglycan, AG10, and AG4/SG6 by WST-1 assay—10% (*v*/*v*) DMSO was used as a positive control.

As a biomaterial for drug delivery, the biocompatibility of agarose/succinoglycan hydrogels would be significant. Cytotoxicity, an important factor in measuring the biocompatibility of the hydrogel, was tested by HEK 293 cells [40]. The cell viability was 98% when treated with succinoglycan and 91% and 103% when treated with AG10 and AG4/SG6 hydrogel, respectively (Figure 9). Dimethyl sulfoxide (DMSO) was used as a positive control for cytotoxicity, and 23% of cells are viable under these conditions. The obtained results demonstrated the non-toxicity of the AG4/SG6 hydrogel on HEK-293 cells, suggesting that the agarose/succinoglycan hydrogels possess good biocompatibility and could be future biomedical materials applied in drug delivery systems.

## 4. Conclusions

In this study, succinoglycan was successfully introduced into agarose hydrogels by a simple method, and the various properties of the agarose/succinoglycan hydrogels were systematically investigated. The agarose/succinoglycan hydrogels improved flexibility and swellability. They also provided pH-responsive properties for effective drug delivery. The rheological results showed that an increase in the succinoglycan proportion in the agarose/succinoglycan hydrogels could significantly enhance the flexibility and swelling property of the hydrogels. ATR-FTIR, FE-SEM, and XRD also confirmed the homogeneous and porous network of the hydrogels, probably due to the interactions between succinoglycan and agarose in the hydrogels. The release of ciprofloxacin (CPFX) from the agarose/succinoglycan hydrogels was significantly dependent on the pH of the medium. They showed a pH-responsive delivery of CPFX, which exhibited a cumulative release of ~41% within 35 h at pH = 1.2 but complete (100%) release at pH = 7.4. Furthermore, agarose/succinoglycan hydrogels were non-cytotoxic, suggesting that they would have great potential as drug delivery systems for biomedical applications. 

## Figures and Tables

**Table 1:** Hydrogel compositions of various agarose and succinoglycan proportions.

Hydrogels	Agarose (mL)	Succinoglycan (mL)	Agarose (%, *w*/*v*)	Succinoglycan (%, *w*/*v*)
AG10	10	0	2	0
AG8/SG2	8	2	1.6	0.4
AG6/SG4	6	4	1.2	0.8
AG4/SG6	4	6	0.8	1.2
AG2/SG8	2	8	0.4	1.6

## Data Availability

Not applicable.

## Supplementary Materials

The following are available online at https://www.mdpi.com/article/10.3390/polym13132049/s1, Figure S1: (a) SEM image of agarose gel, (b) and (c) EDS mapping image for C and O, (d) Area EDS spectrum of C and O; Figure S2: (a) SEM image of AG8/SG2 hydrogel, (b) and (c) EDS mapping image for C and O, (d) Area EDS spectrum of C and O; Figure S3: (a) SEM image of AG6/SG4 hydrogel, (b) and (c) EDS mapping image for C and O, (d) Area EDS spectrum of C and O; Figure S4: (a) SEM image of AG4/SG6 hydrogel, (b) and (c) EDS mapping image for C and O, (d) Area EDS spectrum of C and O.
