# Supplementary material for: Fabrication of Flexible pH-Responsive Agarose/Succinoglycan Hydrogels for Controlled Drug Release
Source: Polymers (Basel). 2021 Jun 22;13(13):2049. doi: 10.3390/polym13132049 (PMC8272162; doi:10.3390/polym13132049)
Supplement: Supplementary file 1 [file polymers-13-02049-s001.zip › polymers-1261451-supplementary.pdf]

Supplementary Materials

Table of Contents

Energy-Dispersive Spectroscopy (EDS) Analysis

Agarose/succinoglycan hydrogels were measured by scanning electron microscopy (SEM) equipped with Energy selective Backscattered (EsB) detector. The samples were coated with gold (Au) using coating system. EDS spectrum directly revealed the presence of the C and O elements in the sample.

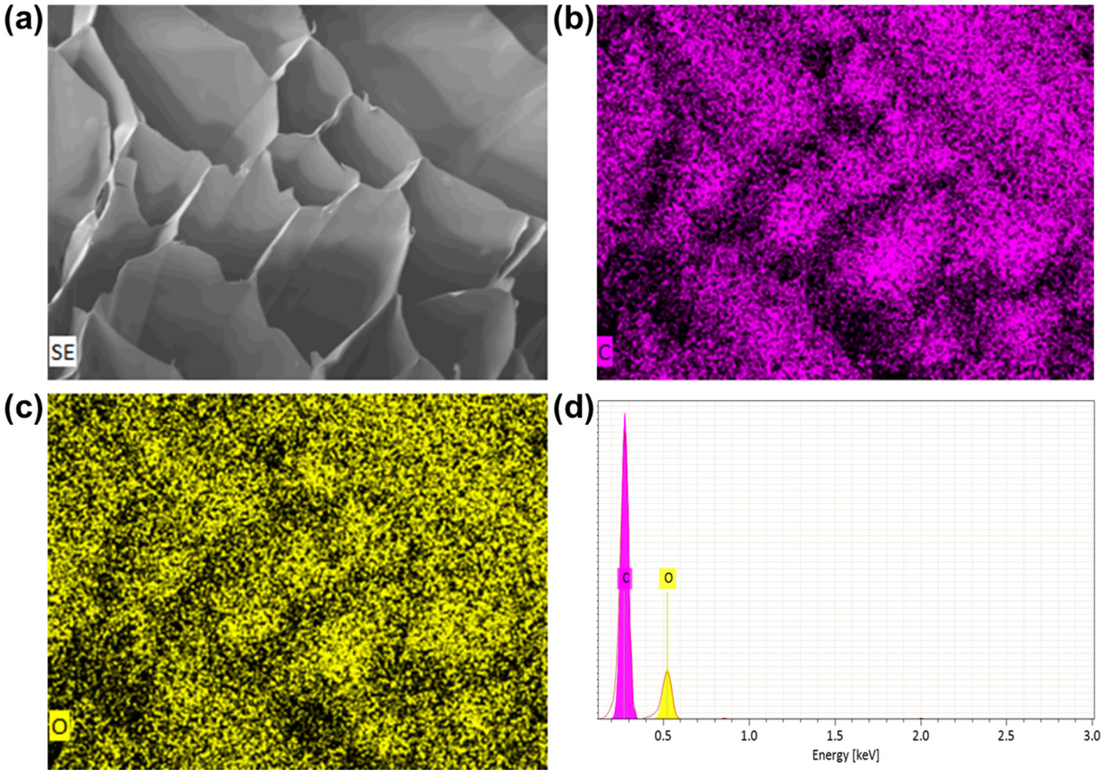

Figure S1. (a) SEM image of agarose gel, (b) and (c) EDS mapping image for C and O, (d) Area EDS spectrum of C and O.

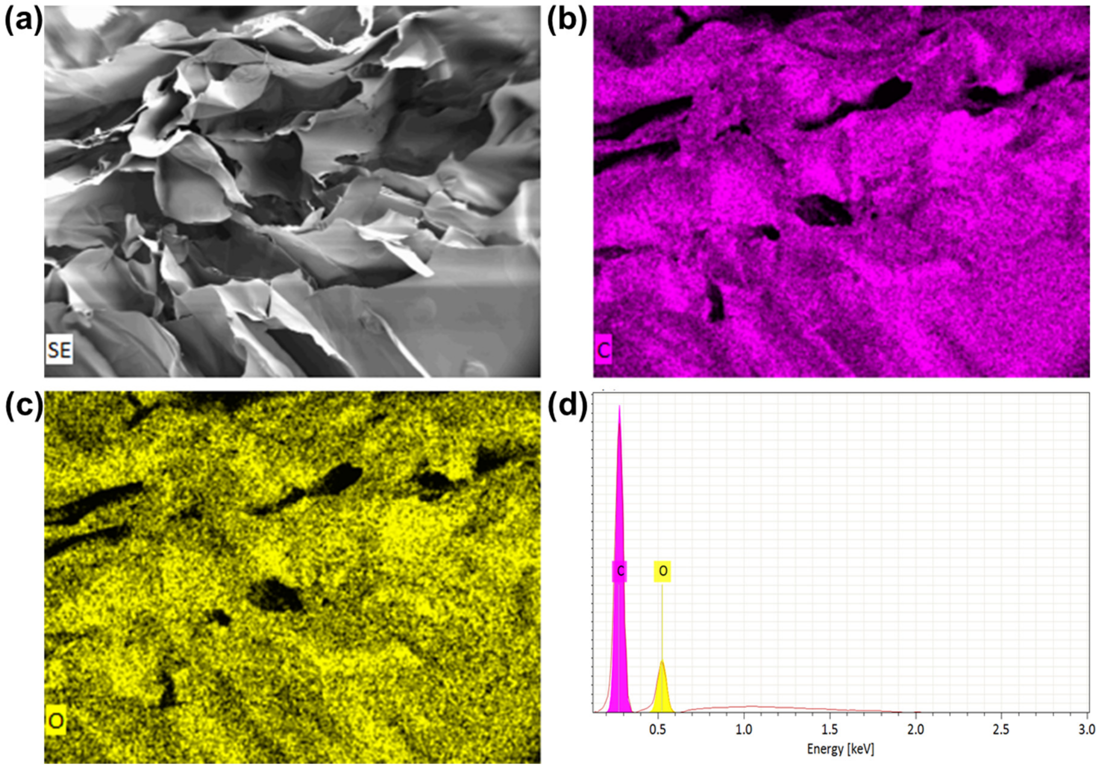

Figure S2. (a) SEM image of AG8/SG2 hydrogel, (b) and (c) EDS mapping image for C and O, (d) Area EDS spectrum of C and O.

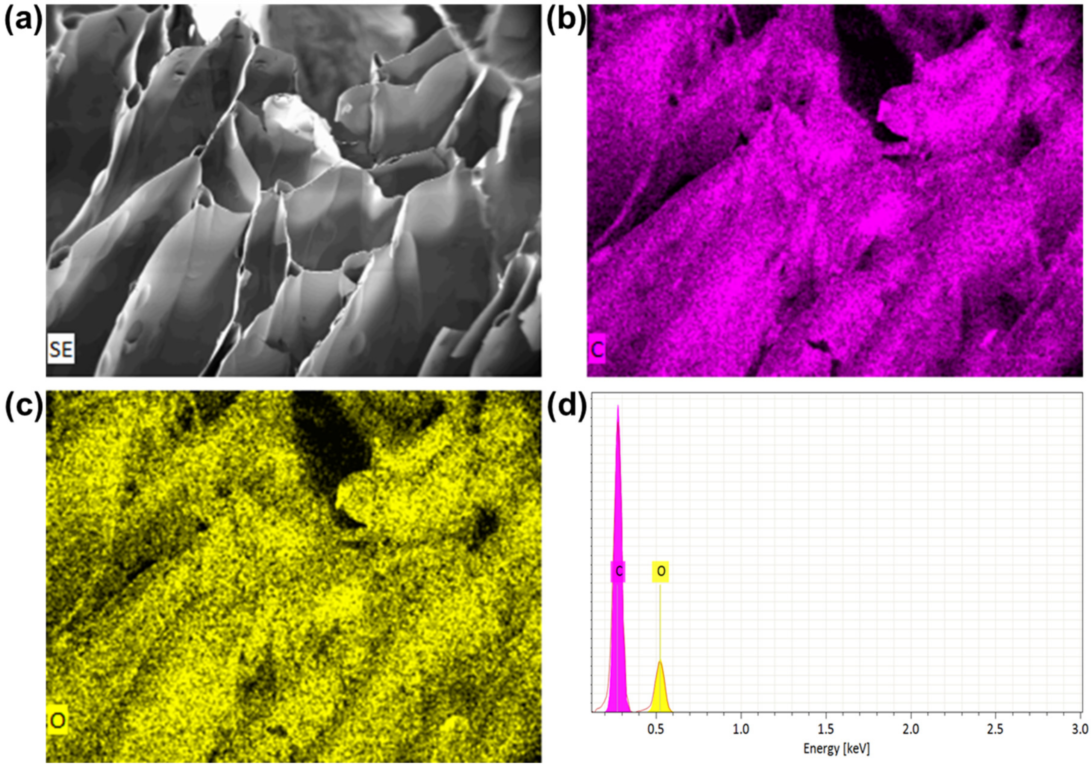

Figure S3. (a) SEM image of AG6/SG4 hydrogel, (b) and (c) EDS mapping image for C and O, (d) Area EDS spectrum of C and O.

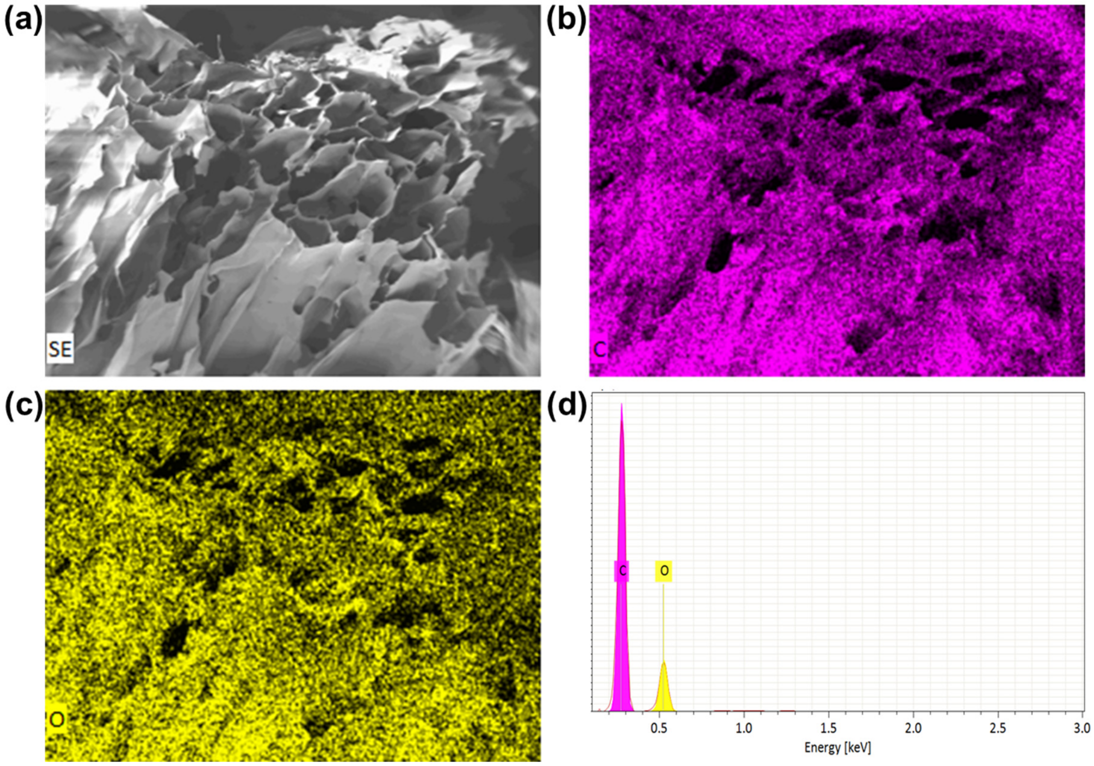

Figure S4. (a) SEM image of AG4/SG6 hydrogel, (b) and (c) EDS mapping image for C and O, (d) Area EDS spectrum of C and O.
